# “Feeling Trapped in Prison” Due to the COVID-19 Pandemic: Perceptions and Practices among Healthcare Workers and Prison Staff from a Brazilian Maximum Security Unit

**DOI:** 10.3390/healthcare11172451

**Published:** 2023-09-01

**Authors:** Wanessa Cristina Baccon, Maria Aparecida Salci, Lígia Carreira, Adriana Martins Gallo, Francielle Renata Danielli Martins Marques, Carlos Laranjeira

**Affiliations:** 1 Departamento de Pós-Graduação em Enfermagem, Universidade Estadual de Maringá, Avenida Colombo, 5790—Campus Universitário, Maringá 87020-900, PR, Brazil; wanessabaccon@hotmail.com (W.C.B.); masalci@uem.br (M.A.S.); ligiacarreira.uem@gmail.com (L.C.); adriana.gallo@ifpr.edu.br (A.M.G.); franrenata.martins@gmail.com (F.R.D.M.M.); 2 School of Health Sciences, Polytechnic of Leiria, Campus 2, Morro do Lena, Alto do Vieiro, Apartado 4137, 2411-901 Leiria, Portugal; 3 Centre for Innovative Care and Health Technology (ciTechCare), Campus 5, Polytechnic University of Leiria, Rua de Santo André-66-68, 2410-541 Leiria, Portugal; 4 Comprehensive Health Research Centre (CHRC), University of Évora, 7000-801 Évora, Portugal

**Keywords:** prisons, prison personnel, Brazil, COVID-19 pandemic, occupational health

## Abstract

The COVID-19 pandemic had several repercussions on prison staff, but the currently available evidence has mainly ignored these effects. This qualitative study aimed to understand the impact of COVID-19 on the prison system through the narratives of health and security professionals, using the methodological framework of the constructivist grounded theory proposed by Charmaz. The sample included 10 healthcare workers and 10 security professionals. Data collection took place between October and November 2022 through individual in-depth interviews. The data were analyzed using the MaxQDA software. Three categories of interrelated data emerged: (1) “Confrontation and disruption” caused by the COVID-19 pandemic in the prison system; (2) “Between disinfodemic and solicitude” referring to the tension between information management and the practice of care centered on the needs of inmates; and, finally, (3) “Reorganization and mitigation strategies during the fight against COVID-19”. Continuous education and the development of specific skills are essential to enable professionals to face the challenges and complex demands that arise in prison contexts. The daily routines professionals had previously taken for granted were disrupted by COVID-19. Thus, investing in adequate training and emotional support programs is crucial to promote the resilience and well-being of these professionals, ensuring an efficient and quality response to critical events.

## 1. Introduction

The COVID-19 pandemic has expanded and emphasized the multiple threats to public health and human rights that have long been associated with the prison system [1]. Prisons around the world were identified as high-risk environments for the spread of the disease [2]. According to estimates, incarcerated individuals have an infection rate that is 5.5 times higher than that of the general population [3]. This rate stems from several factors, including overcrowding of prison facilities, ineffective ventilation, unsanitary conditions, shortage of human resources, and high professional turnover [2,4]. Moreover, prisons house people with several chronic comorbidities [5]. 

With more than 11.5 million people imprisoned worldwide [6], the overcrowded conditions in prisons make it difficult to apply health control measures, in many cases violating human rights. Brazil has one of the largest prison populations worldwide, with over 835,000 imprisoned people [7]. Individuals are housed in filthy conditions, with poor provision of basic necessities, along with inadequate delivery of basic and essential health care, contributing to the rapid spread of the disease [8,9]. To protect people who live or work in detention facilities, the World Health Organization [10] has highlighted the importance of implementing strategies to control the spread of the coronavirus in these institutions.

The system’s response to the COVID-19 crisis reflects the Foucauldian “principles of segregation, segmentation, and surveillance” [11]. The measures implemented by prison authorities to address the COVID crisis illustrate the transformation of prisoners into ‘docile bodies’ [12] (p. 136). These measures include physical distancing between inmates, adequate isolation of suspected and confirmed cases, quarantine, compassionate release (based on their offense background), suspension of visits, and transfers of detainees between units [12]. Foucault argues that disciplinary power operates through the control and regulation of bodies and individual conduct. In this sense, fear, perceived risk, and safety concerns regarding the virus seem to be directly linked to engagement in preventive behaviors [13,14].

Other measures to prevent the spread of the virus were also fundamental, such as protocols for the regular disinfection of the prison’s physical spaces and the use of personal protective equipment, carrying out large-scale tests, providing information and psychological support, and prioritizing the vaccination campaign [15,16,17]. According to Vella et al. [18], the implementation of the vaccination plan was effective in preventing and protecting both the inmates and healthcare staff. Although inmates, the prison healthcare professionals and other workers were priority targets for vaccination, the former received less timely access.

Prison staff were essential during the pandemic, having to adapt to changes in the prison system and assume new responsibilities [19]. This can cause confusion and distress, as there are different expectations and demands to be met [20]. Due to their difficult jobs, hostile working environments, and exposure to both direct and indirect stress, prison staff have poor physical and mental health results. This may make them susceptible populations [21,22].

Likewise, prison staff is disproportionately at high risk for COVID-19 consequences in prison facilities since they move between correctional institutions and their communities [23,24], and this may be exacerbated by additional occupational concerns. Many professionals reported signs of burnout, as distress in the work environment can negatively affect the team’s attitudes and, thus, affect the quality of health care [22]. Prison staff faced additional barriers to adequately meeting the needs of inmates due to “legacy factors” in the prison system [25], including the state of prisoners’ health before the pandemic, and their vulnerability to COVID-19. The fact that they deal with a high-risk population generates greater fear of the impact of SARS-CoV-2 compared to other groups [26]. In addition to their responsibility to preserve the health and safety of detainees, who are considered a vulnerable group [27,28], they should ensure compliance with preventive measures inherent to their function [17], generating high pressure and professional wear.

The main health concerns and dangers related to COVID-19 from a transmission and containment perspective are covered in the most recent evidence on COVID-19 and prisons [29]. However, there are additional, less common discussions regarding the effects of disadvantages and inequities faced by people working in prison settings, which go beyond those intrinsic challenges of prison as a “total institution” dealing with the pandemic [30].

Although there is some qualitative research available [22,31,32], there is a dearth of studies in Brazil looking specifically at the impact of the pandemic on the prison system through the eyes of healthcare and security personnel. 

The present study aimed to understand qualitatively the impact of COVID-19 on the prison system through the perceptions and practices of health and security professionals. In this sense, the research question was: How do healthcare and prison staff perceive and deal with the impact of COVID-19 in a Brazilian high-security prison? We hope this study creates a robust field of knowledge and generates guidelines for the implementation of effective measures capable of dealing with future catastrophe scenarios.

## 2. Materials and Methods

### 2.1. Study Design

This study is part of a larger research project aimed at assessing the effects of the pandemic on the prison system in the state of Paraná, Brazil. The present study used a Constructivist Grounded Theory design (CGT) based on the principles of Kathy Charmaz [33]. This approach seeks to understand how individuals construct their realities and focuses on the interpretation of the meanings attributed by the subjects. It values the interpretive perspective of the participants and seeks to explore the processes by which meanings are constructed and shared [33]. 

This study was conducted and reported following the Consolidated Criteria for Reporting Qualitative Research (COREQ) checklist [34]. 

### 2.2. Setting, Participants and Recruitment 

The study was carried out in a maximum-security prison with a capacity for 960 male persons located in the municipality of Maringá—northwest of the State of Paraná—in Brazil. The facility was built with the initial objective of only receiving individuals who were awaiting trial, but, due to the shortage of vacancies in other prisons, it ended up receiving convicted individuals. This choice was made because this is one of the largest prison facilities in one of the Brazilian states most affected by the COVID-19 pandemic [35].

The participants were included incrementally, according to the principles of CGT [33], first by purposeful selection and then through a focused theoretical sampling approach.

A maximum variation purposive sample was used to guarantee a heterogeneous sample in terms of age and professional experience. Participants were healthcare and security professionals with a strategic role in the prisons during the pandemic. Study eligibility depended on the following criteria: (a) work experience in a prison before the onset of the COVID-19 pandemic; (b) preset format work during the data collection period; and (c) ability to understand and communicate in Portuguese.

The theoretical sample included 10 health professionals and 10 security professionals (prison officers) who voluntarily participated in the in-depth qualitative interviews. This number was determined by theoretical saturation, as recommended by Charmaz [30]. 

### 2.3. Data Collection 

Data collection was conducted over 2 months (from October to November 2022), for reasons of organization and functioning of the unit. We identified target participants through the research team’s professional network. Before signing a consent form, those who manifested interest in the research were given a participant information leaflet.

The interviews were carried out by only one nurse, with large clinical activity in prison facilities (W.C.B.). A semi-structured interview guide prepared by the researchers and based on available evidence was used as an instrument for data collection [17,21]. The interview guide addressed the following topics: perceptions and experiences regarding work during the pandemic period; barriers encountered in the performance of their functions; strategies used to deal with these barriers; the availability and access of prisoners to health services; and, finally, how professionals have changed moving forwards.

Before starting data collection, the instrument was validated by three experts with PhDs and experience in qualitative research, who validated the content, form, and clarity of the questions, obtaining an agreement level of greater than 90%.

Given that data collection was concomitant with data analysis, the interview script was modified according to emerging themes, in particular, to improve understanding of the phenomenon under study [33]. Field notes were taken during and after the interviews, and memos were written by the researcher to guide the analysis, following the principles of CGT.

The interviews were carried out in a specific place at the institution, which ensured the privacy of the interviews. All interviews were conducted in person. There were no repeat interviews. The interviews lasted between 40 min and 90 min, with an average duration of 60 min, and were recorded in audio and transcribed in full.

In order to ensure the accuracy of the citation translations, they were first translated into English and then back-translated into Portuguese. The sample excerpts were numbered according to the role of the participant, being HealthCare Professionals (HCP) and Security Professionals (SP).

### 2.4. Data Analysis

According to the approach suggested by CGT, an abductive approach was adopted in the analysis and codification of qualitative data [33]. Data analysis was conducted with the support of MaxQDA^®^ Software–version 2018 (VERBI Software, Berlin, Germany) to facilitate data management as well as store it [36].

The data collection process was carried out simultaneously, based on three stages [33]. The first stage was the initial coding, which involved open coding carried out incident by incident. The second stage was focused coding, in which categories and subcategories were formed, and grouped based on their similarities. In this process, the researcher’s perception became relevant to identify the central category, representing a significant step in the research area [37]. The theoretical links between the categories will be more apparent after the selective coding step. The theoretical coding phase of a grounded theory is the next stage, during which more, carefully chosen data is gathered in order to advance the emergent theory and elucidate the key categories that make it up [33]. 

### 2.5. Study Rigor

To guarantee the rigor and trustworthiness of the study, the criteria proposed by Charmaz and Thornberg [38] were adopted: reliability, originality, resonance, and usefulness. Credibility was achieved through various approaches. The interviews were meticulously transcribed, and the field notes were carefully compared and verified, ensuring the fidelity of the collected data. In addition, additional interviews were carried out to confirm the emerging theoretical categories, ensuring the reliability of the findings.

Likewise, triangulation was used as a strategy to validate the evidence obtained. Several sources of data, such as interviews, observation, and review of the existing literature, were used to corroborate the results and strengthen the reliability of the research.

Another relevant aspect was the prolonged involvement in the field. This commitment allowed establishing a relationship of trust with the participants, ensuring that they felt comfortable authentically sharing their experiences. Peer debriefing was carried out throughout the research process [39]. This practice involved members of the research team, which enabled in-depth discussions and constant reflection on the findings and interpretations. In addition, brief memos were prepared together with the project team, allowing for a critical and innovative approach [33,37]. To ensure the resonance and usefulness criteria, during the analysis process, schematic representations were also created to visually describe the study findings.

The researcher’s reflexivity has become increasingly relevant in the context of GT, which values the joint construction of knowledge [33]. This reflective approach contributed to increasing the transparency and credibility of the research process. Throughout the different stages of the study, from topic selection and research question formulation to study design, data collection and analysis, and even the writing process, reflexivity was used to reflect on the researcher’s interactions.

The research team involved a nurse with experience in caring for the prison population and undertaking a Ph.D. in nursing at the moment of data collection (W.C.B.). In addition, the team had researchers with expertise in qualitative health research from a constructivist stance (M.A.S., L.C., A.M.G., F.R.M.M., and C.L.). 

### 2.6. Ethics

The research was conducted under the assumptions of the Declaration of Helsinki and was duly authorized by the Research Ethics Committee of the State University of Maringá—UEM (Opinion No. 3,211,746). Before each interview, written informed consent was obtained from participants, including approval for audio recording. They were duly informed that they had the right to withdraw from the research at any time, without any consequences. It should be noted that participants did not receive any monetary incentive for their participation.

## 3. Results

### 3.1. Sample Description

The sample consisted of 20 participants, 10 of whom are health professionals, with an average age of 44.7 years. The majority of these health professionals are white females (60%), and they have an average tenure in the prison unit of 14.9 years. All participants had a history of COVID-19.

The second sample group is comprised of 10 male security professionals, with an average age of 49.3 years. The majority are white (80%), and they have about 11.4 years of professional activity in prison. Only one prison officer reported having no history of COVID-19. Table 1 shows the characterization of the sample. 

### 3.2. Findings from the Analysis

**Figure 1:**
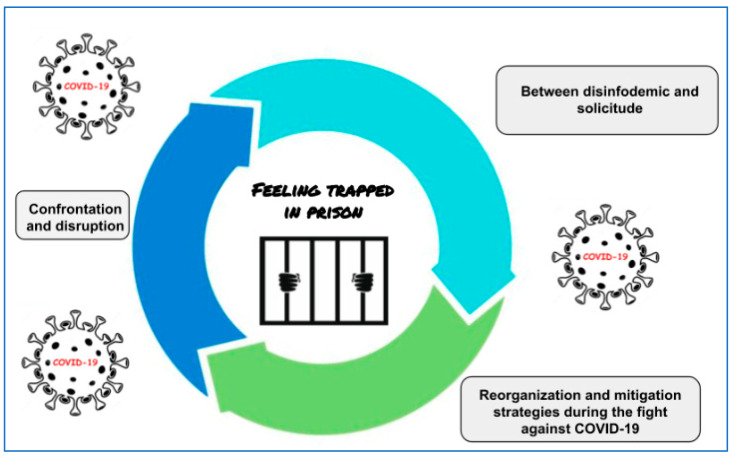
Model of repercussions of COVID-19 on the prison system.

The findings were related through an analytical process formed by three categories: (1) Confrontation and disruption; (2) between disinfodemic and solicitude; and (3) reorganization and mitigation strategies during the fight against COVID-19. The relationships and interactions between the categories were analyzed and a central element that permeates the entire data set was identified. The core category revealed was “Feeling trapped in prison” (Figure 1). Working in a highly restrictive environment, where actions and movements are strictly controlled, can create a feeling of restriction even for those who are responsible for ensuring the health and safety of prisoners.

According to the participants, the changes in routines and the functioning of work activities due to the COVID-19 pandemic resulted in a series of challenges. The fear generated by the spread of the virus combined with the misinformation that was circulating exacerbated the feeling of being trapped in the workplace. The separation between personal and professional life has become increasingly difficult, leading to unregulated schedules and an imbalance between the two domains.

#### 3.2.1. Confrontation and Disruption

This category reveals the confrontation of health and security professionals in the prison system with an unknown reality, which generates manifestations such as fear and emotional dysregulation. The participants faced a complex and restrictive environment, with difficult working conditions, with special emphasis on the initial phase of the pandemic, given the high degree of unpredictability and uncertainty. However, they acknowledge the greater pressure experienced by other structures, such as hospitals. As stated,

*[…] Its start was frightening because it was a new disease with an uncertain evolution, long hospitalization periods, and many cases progressing to death*.

(HCP4)

*It was emotionally draining […] dealing with fear during the pandemic was one of the hardest things. The fact that we don’t know much about the virus and all the resulting complications; deaths happening daily due to the various difficulties health teams faced everywhere*.

(HCP3)

*At first, there was a bit of despair, but then I started following other health professionals, and I realized that our front line wasn’t really that much of a front line. Compared to my friends working in the ICU* (Intensive Care Units), *hospitals, and UPAS* (Emergency Care Units), *so I think that, relatively, it was not as difficult as what was faced at the entrance doors*.

(HCP6)

Prior knowledge about infectious diseases made the participants fear both personal contamination and the possibility of transmitting the virus to their loved ones, reducing contact with the outside world.

*Knowing the impact that an infectious disease has, the biggest concern was taking it to our families, so we reduced the number of trips home […] when the initial fear subsided, there was a concern about elaborating or trying to minimize the impact of the pandemic*.

(HCP2)

*It was difficult, I won’t say it was easy, I had to control what I would take home, it was complicated, I was afraid, I had already been infected and I was afraid. In September, I had a bad time, I didn’t go to the hospital, I was afraid to take it back home, and it was complicated*.

(SP5)

Given the unprecedented character of the pandemic and the structural limitations, the participants underline the difficulty of identifying the chains of infection within the prison. Their share of responsibility for the transmissibility of the disease was recognized, as they were the ones who entered and left the prison unit daily.

*The agents of the penal system were the main vehicles of transmission because they left the prison daily, so when cases arose, it was because they brought it from the community*.

(SP9)

*The prison officers were the ones who brought the disease into the prison*.

(SP4)

Some security professionals perceive the prison context as more dangerous concerning the spread of the virus compared to other community contexts. In this regard, SP9 mentions: “*[…] the virus was contagious, but it didn’t seem as aggressive outside as it did inside the system […]*”. This perception may be related to the specific challenges experienced within the prison system, such as the difficulty of adequate physical distancing, the scarcity of resources, and limited access to health care.

*[…] Human resources are needed. We don’t have enough human resources to provide a quality health service*.

(HCP2)

*The health area is very precarious […] we have no voice, we are not recognized or even remembered*.

(HCP9)

*[…] we have a big shortage of employees, […]* (the institution) *should hire more people*.

(HCP1)

The difficulty in finding cells inside the prison for the isolation of positive cases was identified as a challenge for the participants. Given that overcrowding is a recurring problem, there is a regular demand for cells to accommodate a large number of inmates. In this sense, finding space for isolation cells created significant disruption.

*[…] all incoming prisoners, even those without COVID-19, had to be quarantined, and we didn’t have space for that, and that created a certain operational difficulty*.

(SP7)

With the implementation of protocols for the care of suspected or confirmed cases of COVID-19, participants reported a sense of unpredictability in the face of the evolution of the pandemic in the prison system. This fact determined that new information and guidelines were constantly emerging. Added to this is the lack of prior experience in dealing with a pandemic of this magnitude and the need to adapt quickly to changes. At the same time, the complexity of the prison environment also exacerbated feelings of uncertainty among all those involved on the front lines. In several cases, working hours were extended to respond to needs, and sometimes feelings of detachment from the outside reality arose.

*We made decisions according to protocol […], but always with a feeling of uncertainty about everything we did. Whether it was good or not, I don’t know […] but at the same time, it relieves me, seeing that other places have gone through the same situation, where the problem was the same*.

(HCP6)

*Non-health professionals were always asking questions, wanting answers […] some new behavior raised questions, everything was new. Behaviors changed from one day to the next and we had to adapt*.

(HCP5)

*We had to work long hours, which was very complicated because we didn’t see the family*.

(SP1)

#### 3.2.2. Between Disinfodemic and Solicitude

During the COVID-19 pandemic, fighting the disinfodemic and showing concern were recognized as key strategies to ensure the safety and well-being of inmates, as well as the prison staff involved.

A disinfodemic describes the large-scale spread of false, unreliable, or misleading information. Health professionals faced the challenge of dealing with a significant volume of information about COVID-19. Therefore, they had to adopt strategies such as the active search for reliable sources and the constant updating of scientific knowledge. In the initial phase of the pandemic, there was concern among workers about the misinformation associated with COVID-19, which generated high skepticism. In parallel, participants report having responsibility for filtering and disseminating accurate and up-to-date information, with the aim of promoting an adequate understanding of the disease and preventive measures among inmates and their families.

*In the beginning, it was a new experience, but as the days went by, it brought fear and insecurity, culminating in skepticism […] I didn’t believe in anything anymore*.

(HCP10)

*We had a large amount of information, much of it inaccurate. We serve as multipliers of information not only in our functions but also to the prison population and staff. We experience difficulties with several misguided guidelines without scientific basis that have been released*.

(HCP4)

Health professionals point out a limitation, the lack of communication during the pandemic. Several factors may have had an impact on the capacity to respond and manage the health crisis in prisons, causing delays in the dissemination of information, such as health guidelines, safety protocols, and updates on COVID-19 cases.

*There are no meetings to discuss or talk about assistance, standardization of conduct, or procedures with the security team and the health sector*.

(HCP4)

The exchange of information and moments of debriefing between colleagues was evaluated as a positive aspect of managing day-to-day situations in the pandemic context. This collaboration allowed professionals to share experiences, coping strategies, and up-to-date knowledge on disease management in a challenging environment. This interaction and collaboration between colleagues contributed to improving the care provided to prisoners and to strengthening the health team in facing the pandemic in a prison context.

*I can say that crucial facilitating aspects in this context were the support and information shared with/among co-workers. They were fundamental*!

(HCP9)

*The moments of sharing between peers were important, I see more in that sense, the positive points*.

(HCP2)

*We stayed up late, discussing what could be done even more in an environment that is conducive to the proliferation of these microorganisms and we work directly with confined people, a higher-risk environment. Our concern was mainly to try to reduce the effects here*.

(HCP2)

In many cases, prisoners’ access to the health sector within the prison units went through the scrutiny of prison agents. These professionals usually act as intermediaries between prisoners and health professionals. In this context, solicitude emerges as a possibility of openness and acceptance of the other through an intersubjective relationship that provides attention and care.

*Our role is to be the link because one thing they* (inmates) *usually say is that we are their hands and feet, so we try to have a good relationship. When there is good contact and mutual respect, this favors the relationship*.

(SP2)

The participants’ concern to promote monitoring and the creation of an environment conducive to dialogue is highlighted, particularly at times when inmates adopt extreme measures to make themselves heard. Different forms of protest were mentioned, such as making noise, shouting, banging on the railings, or even starting riots to draw the attention of the authorities and demand medical attention.

*At certain times they* (inmates) *start to get excited, so the tendency is to knock, shout, kick, to knock on doors. They think that by doing this, they will speed up the process, but sometimes they don’t […] that’s how they do it*.

(SP2)

#### 3.2.3. Reorganization and Mitigation Strategies during the Fight against COVID-19 

The reorganization and implementation of COVID-19 mitigation strategies in prisons was a complex process due to the specific characteristics of this environment. The closed nature of prisons and close contact among inmates has posed challenges in preventing the spread of the virus.

Health professionals highlight the concern with the underreporting of cases in the prison system. Additionally, they mention that there is a lack of adequate and accurate records of COVID-19 cases within prisons, which may lead to an underestimation of the real extent of the disease in these institutions.

*[…] The doctor did not frequently ask for the screening test. In the UPA* (Emergency care unit) *all people are tested. She (the doctor) tests one case or another, the most serious, but it is not transverse conduct for all symptomatic inmates*.

(HCP6)

Participants evaluate virus mitigation strategies as positive points in prisons. They further recognize that the implementation of these measures was crucial to reducing the risks of spreading COVID-19 and ensuring the safety of both inmates and staff.

*There was a greater demand among employees for prevention and this was a positive point*.

(SP10)

*The changes were effective, I believe so […] they made it much easier for the virus not to spread*.

(SP3)

*I believe that some measures were effective in reducing exposure to the SARS-CoV-2 virus, however, the administration should have provided masks with greater protection capacity*.

(HCP2)

The compassionate release of prisoners was also a measure to mitigate the virus, particularly for individuals with greater vulnerability to COVID-19, but always depending on the type of crime committed. HCP6 mentions: *[…] one of the strategies was the departure of inmates who had some comorbidity. It was very complicated […] because there are crimes that could not go home*.

Awareness of maintaining hygiene standards even after the COVID-19 pandemic is an important aspect highlighted by the participants. Although the pandemic has increased the need for hygiene measures and precautions to prevent the spread of the virus, it is necessary to maintain these practices in the long term.

*It is important to continue using a mask, especially when there is a risk of infection, just adjust the use of a mask, alcohol gel, and gloves*.

(SP8)

*We’ve already lost a lot of people […], we have to learn from what happened. And we must be careful, wear a mask, and get vaccinated, or we run the risk of coming back again*.

(HCP1)

The implementation of stricter screening measures for the early identification of suspected cases, increased testing of inmates and staff, increased access to Personal Protective Equipment (PPE), and specific training on prevention and response to COVID-19, in addition to establishing an efficient flow of communication, were strategies adopted to mitigate the virus.

*We had several strategies, we adopted protocols within the prison units, and we had a lower mortality rate because, through DEPEN* (National Penitentiary Department), *some ordinances determined the carrying out of screening tests and prohibited, for example, visits, the delivery of food and hygiene goods*.

(HCP2)

*We produced fabric masks for the inmates and the agents, aprons and caps for us and for the hospitals […] the PPE was in short supply everywhere, all the stock was running out, and that was one of the strategies that we also had to invent*.

(HCP6)

*We implemented continuous education to create moments of discussion and training with prison agents. In the first phase of the pandemic, we were concerned with clarifying the doubts of security professionals*.

(HCP2)

Some professionals noticed improvements in resources and processes within the prison system resulting from the measures implemented due to COVID-19. Emphasis on the availability of gel alcohol dispensers in strategic locations, the expansion of the supply of hygiene products, the implementation of cleaning protocols, and awareness of the importance of personal hygiene. These changes were seen as positive, as they contributed to the promotion of a healthier environment.

*The placement of alcohol dispensers in the corridors, in strategic locations, and in the toilet, to wash hands, has increased significantly. It was even good because we have always wanted it, but we didn’t have it. Then, liquid soap and paper towels to dry hands started to appear, which was something very scarce*.

(HCP6)

*The prison environment is somewhat unsanitary, so hygiene measures ended up making the environment cleaner*.

(SP7)

Likewise, vaccination against COVID-19 brought peace of mind to the professionals involved, considerably reducing the risk of contamination and the possible serious effects of the disease.

*Encouraging people to take the vaccine and booster doses became my main focus […] receiving materials for the rapid COVID tests and vaccine doses has positively eased the policy of social isolation*.

(HCP10)

*[…] and after the vaccine things got easier […] and the prisoners joined in*.

(HCP1)

Professionals also mentioned that by restricting visits it was possible to reduce the likelihood of the virus entering the prison environment.

*Regarding facilitating actions, the cancellation of visits, quarantines after leaving the unit for care, and the creation of a sentinel unit in suspected or confirmed cases of COVID-19*.

(PS4)

*The inmate was left without a visit and isolated and that was positive to control and not turn the pandemic* (in prison).

(PS7)

In this sense, virtual visits were instituted as an alternative to the suspension of face-to-face visits. Perceptions about virtual visits varied, ranging from recognizing their importance in guaranteeing the proximity of family ties to expediting the holding of court hearings, despite the lack of training and digital literacy in their use.

*Online visits were positive, it worked well. The system already existed and was perfected. Before, the connection was precarious, but it has improved, court hearings with video were also a gain*.

(SP5)

*[…] The system of visits by videoconference was initiated without any training or IT support being offered to the professionals who would carry out this task. This was detrimental to overworked professionals and also to visitors and prisoners*.

(HCP8)

*Videoconferencing visits helped a lot because there was no contact between people*.

(SP8)

Participants have the perception that prison is a complex environment permeated by prejudices and stigma, which determines the need for a holistic and humanized approach, capable of considering individual particularities, with a view to their reintegration and resocialization.

*[…] here is not a bunch of people […] society thinks that the person is arrested and disappears […] the prisoner will continue eating, living, needing medical attention, that’s why the State has to wake up and see that one-day people will get out… whether they will leave “worse” or “better”, depends on the available staff and structure*.

(SP9)

*[…] We value doing our best, we refrain from passing judgment. As representatives of the State, we go and find solutions*.

(SP8)

## 4. Discussion

In this study, we aimed to understand the impact of COVID-19 on the prison system through the narratives of health and security personnel. These narratives cast doubt on widely held notions about the effective control of COVID-19 in prison environments and clarify the problems and obstacles the pandemic has presented prison staff and inmates. Like previous studies, participants showed fear of contracting COVID-19 and of being vehicles of transmission, putting their family members at risk [8,40].

Fear can have an undesired effect, generating emotions of powerlessness that can lead to defensive responses instead of an active approach [17]. Additionally, excessive fear can be harmful to mental health, manifesting in symptoms such as anxiety, stress, depression, and other psychological problems [41].

Participants in our study pointed out uncertainties regarding COVID-19, which according to Liu Ye [9] reflects the need for more protective measures to ensure safety in the workplace. The existing concern highlights the need to improve precautions to provide a safer work environment.

Although prison staff and inmates are subject to measures of varying complexity instituted to control COVID-19, they were all required to follow the same guidelines in the use of Personal Protective Equipment (PPE) and social distancing, reflecting a regulatory uniformity imposed by institutional power. This observation is related to the ideas of Michel Foucault, who discussed the concept of biopolitics [13], which refers to the use of state power to regulate and control the life of the population, including in terms of health and well-being. In this sense, health guidelines during the pandemic can be understood as a biopower mechanism, exercising control over the lives of prisoners and staff [13]. The findings highlight how COVID-19 disrupted previous roles and identities, challenging the power dynamics established in the prison environment as discussed by Foucault [42,43]. This situation has led to the emergence of new relationships and interactions between staff and inmates, with potential changes in the power structure.

During the pandemic, important challenges were highlighted by prison staff due to the high labor demands and pressures [4,44]. Managing and promoting a positive work environment plays an important role in improving burnout and compassion fatigue, as they offer an empathetic lens to help employees deal with stressors associated with the health crisis. In this regard, studies reveal moderate levels of burnout and compassion fatigue, as well as difficulties in maintaining satisfaction with compassion for professionals working in a prison context [44]. This situation was aggravated by the pandemic crisis, where levels of anxiety and burnout increased [45].

Although participants generally recognized they were capable of protecting themselves against COVID-19 in the workplace, some mentioned working in unhealthy conditions, perceiving a negative impact on mental health brought about by issues related to the pandemic, and frequent concerns about the possibility of becoming infected in the workplace and taking the virus from the prison into their personal environment [23].

Foucault argues that power relations are not limited to the scope of law or violence, nor are they limited to contractual or merely repressive aspects. According to him, power operates as a network in constant circulation, where individuals are not only passive or consenting targets but also centers imparting this power [13,46]. This conception of networked power is relevant to understanding how prison staff dealt with the challenges of the pandemic. Amid constant changes in the environment to prevent the spread of the virus, they came together and adopted safety strategies, demonstrating an evident concern with the unique consequences of this experience [4]. 

In this context, the shortage of PPE, the difficulties in maintaining physical distance, and the lack of consolidated information about the necessary care in relation to the disease, reveal the limitations and challenges faced by the staff. This situation generated anxiety among the staff since they were working in an environment where most people were advised to stay at home selves [4]. The interaction between power, care, and life control strategies in the prison environment during the pandemic highlights the relevance of Foucauldian concepts to understanding the dynamics present in this context.

Foucault [47] highlighted the interconnection between power and self-care, emphasizing how power structures can influence the way individuals perceive and care for themselves. In this context, prison staff faced obstacles to ensuring their health and well-being while facing the demands and pressures of the prison environment during the COVID-19 crisis [4].

Health and security professionals faced difficulties when handling the detainees’ demands. Prison officers perceived themselves as spokespersons for the detainees within the prison, but often the dialogue between the parties was not satisfactory. The findings of this study must be interpreted considering the broader power dynamics that occur in prison institutions [43]. The employee-inmate division and the perceived distance between these two roles are challenged when individuals assumed different roles or when similarities between groups are identified [42]. The COVID-19 pandemic fostered this division between staff and detainees, upsetting the previously existing balance between these two groups [42].

As in our findings, the literature indicates the possibility of underreported COVID-19 cases in prisons [9,48,49], due to the lack of adequate testing among inmates and prison staff. Evidence underlines that mass testing has proven to be highly effective in containing the spread of COVID-19 in a prison environment [16]. Participants in the current study revealed concern about the lack of prisoner testing, a situation also identified by Liu [9]. This perception seems to reflect the existing distrust in institutions or healthcare professionals, hindering the pursuit of proper care and adoption of preventive measures, such as vaccination.

The inmates’ distrust of the health care they receive can be understood as a manifestation of the disciplinary power that permeates the prison environment, where the surveillance and control exercised by the staff can result in a perception of negligence or lack of care [43]. In contrast, professionals had a higher level of exposure to the virus, as they maintained contact between the prison environment and the community [50]. This exposure justified subjecting them to mandatory tests. Evidence revealed a significantly higher prevalence of infection among professionals when compared to inmates [16,23].

Although the need to implement measures to control the virus within prisons is widely discussed, professionals reported difficulties in following these guidelines and implementing protocols. These guidelines include structural changes, such as reducing the number of inmates, cells for preventive isolation, and improving ventilation to reduce the potential for transmission in prisons. In addition, efforts to increase vaccination coverage were considered important, especially among prison officers [51].

Given the resource constraints, some participants expressed concerns about the acquisition of personal protective and hygiene materials, such as masks, gloves, and other essential items. Faced with this situation, professionals took the initiative and the inmates dedicated themselves to the production of protective equipment for local prison institutions, in addition to supplying materials to hospitals. Other studies have reported this difficulty faced by inmates, who often improvise their own personal protective equipment, highlighting the importance of solicitude and mutual care among these people [52].

To mitigate the impacts of the pandemic on the prison system, drastic measures were implemented to eliminate or reduce COVID-19 infections in prisons. These measures included the early release of incarcerated individuals, especially those at higher risk of serious complications due to COVID-19; the suspension of social or legal visits to detained persons; the quarantine of newly incarcerated individuals; and the reinforcement of hand hygiene [23]. This study’s participants also mentioned these measures.

It is necessary to direct resources toward alternatives to incarceration and approaches that prioritize the health and well-being of individuals, recognizing that the prison system poses significant challenges to the protection of public health and guaranteeing respect for human rights [5].

Our findings suggest that the shortage of qualified professionals is a significant obstacle, representing one of the greatest challenges to containing the spread of COVID-19 in prisons [5]. In line with other studies [23,53], the lack of a primary healthcare team in prison institutions was identified as the main difficulty, resulting in unnecessary escorts and security threats.

Misinformation about COVID-19 has also been prolific, threatening not just individuals but societies as a whole. This leads people who ignore scientific advice to put themselves in harm’s way, widens the lack of confidence in politicians and governments, and diverts the media’s efforts, which work reactively to refute untruths rather than proactively producing information from new data. In this sense, and similarly to other studies [54], our findings underscore the need for monitoring and countering misinformation responses (peer debriefings, dissemination of information from credible sources) that help identify, demystify, and report misinformation about COVID-19. In contrast with policies focused on punishing or rehabilitating inmates, policies focused on lowering COVID-19 risks do not explicitly aim to reduce crime. Instead, they represent compassionate criminal justice policies, whose main goal is to enhance the welfare of detainees [55].

The performance of health professionals in the elaboration of mitigation strategies is important since their knowledge and experience are fundamental for the collective construction of knowledge during the COVID-19 pandemic [56]. In this sense, Foucault [43] asserts there is an interconnection between power and knowledge, wherein power is not limited to hierarchical structures but is also present in knowledge relations and in the production, distribution, and use of knowledge.

### 4.1. Strengths and Study Limitations

One of the strengths of the present study was the use of a sample that includes health and safety professionals with varied experiences, providing a comprehensive view of perceptions and practices in the prison context. Furthermore, the qualitative approach adopted in the study allowed an in-depth analysis of the qualitative data so that it resonates with the voices of the participants. Lastly, the study fills a knowledge gap, given that the findings have the potential to inform the implementation of measures that promote better health and safety for incarcerated individuals.

However, some limitations must be considered. One of the limitations of the study is that it only included data from a prison in the south of Brazil, which limits the transferability of the results to other prison contexts. The interviews took place in prisons during the times when correctional personnel were on duty. Due to time constraints, it was not possible to guarantee that the interviews would go without pause. It is important to consider that the study was carried out in a certain period of time (the third year of the COVID-19 era), reflecting the perceptions and practices of professionals during this specific period of the pandemic. Given that the evolution of COVID-19 had different trajectories in different Brazilian states, the findings may not reflect the reality of the prison system at the national level.

It is essential to highlight the need for more research with quantitative and qualitative approaches of a longitudinal nature to deepen the understanding of the impact of the pandemic in the prison context. Future research should also validate our findings including samples with maximum variation in terms of geographic location and type of prison unit.

### 4.2. Implications for Practice

The study presents the challenges faced by health and security professionals in the performance of their duties during the pandemic. Based on these perceptions, it was possible to identify the support needs of these professionals, such as emotional support, additional resources, or specialized training [57]. 

The current study has implications of great relevance for health practice in the prison context. By understanding the complexities and challenges faced by health professionals in this specific environment, it is possible to identify measures and strategies that help to reduce the impacts of the pandemic and ensure the provision of adequate care to incarcerated individuals. This deeper understanding contributes toward improving health practices and allows for a more effective and targeted approach to health promotion and disease prevention within the prison system, where the use of digital technologies can facilitate bringing services closer to the community.

Another important implication of this study is the possibility of developing targeted interventions to address the specific challenges of the prison system during the pandemic. This would allow the development of more effective programs and policies, such as mental health programs, infection prevention education, and improved training in biosecurity measures. Meanwhile, these measures can also reduce other contagious diseases (such as tuberculosis, influenza, or mumps), highly prevalent in prisons [58].

The leaders of a prison organization must consider implementing comprehensive and regular testing policies and protocols, as well as prevention and control measures, to ensure the safety and well-being of inmates and prison staff and prepare for future disaster scenarios. 

Finally, carrying out this in-depth qualitative study contributes to the advancement of scientific knowledge about the pandemic in the prison context. By filling existing knowledge gaps, the study provides a broader understanding of the challenges faced and best practices in coping with the pandemic in prisons. This is fundamental to support future research and guide interventions and policies in the prison system.

## 5. Conclusions

As far as we know, this is one of the first attempts to explore the impact of COVID-19 on the prison system, providing important insights through the lens of prison staff. In the context of the pandemic, research in prison units is a rarely explored field due to the novelty and complexity of the disease.

Prison is, by nature, an environment where one is likely to feel confined. Moreover, qualitative analysis revealed a sense of “feeling trapped” due to several changes associated with the pandemic and the legacy factors that still prevail in prison settings. One of the main aspects highlighted by participants was the fear of contracting the disease and subsequently transmitting it to their loved ones. This fear reflects concern for their own health and the responsibility to protect those in close contact with them. The unavailability of clear and up-to-date guidelines on disease prevention and management protocols in the prison environment made it difficult to make appropriate decisions and effectively implement preventive measures. This generated uncertainties and difficulties in adopting practices that could minimize the spread of the virus among inmates and health and security workers. Another challenge was the shortage of personal protective materials and equipment, essential to guarantee the safety of prison professionals. Likewise, staff clearly struggled to navigate humanity in the prison and solicitude was a necessary condition to respect inmate vulnerability inmates.

## Figures and Tables

**Table 1:** Participant characteristics (n = 20).

Variables	Healthcare Professionals (n = 10)	Security Professionals (n = 10)
**Age** (years)		
Mean ± SD (range)	44.7 ± 4.92 (37–52)	49.3 ± 8.24 (30–58)
**Race**		
White	6	8
Yellow	2	–
Black/African descent	2	2
**Sex**		
Female	6	–
Male	4	10
**Education**		
≤8 years	–	–
≥9 years	10	10
**Profession**		
Clinical Social Worker	1	–
Dentist	1	–
Nurse	4	–
Nurse technician	4	–
Prison Officer	–	10
**Length of service** (years)		
Mean ± SD (range)	14.9 ± 4.77 (9–27)	11.4 ± 6.29 (3–17)
**History of COVID-19**		
Yes	10	9
No	–	1

## Data Availability

All data generated or analyzed during this study are included in this article. This article is based on the first author’s doctoral thesis in Nursing at the State University of Maringá—Brazil.
